# 2.5D MFFAU-Net: a convolutional neural network for kidney segmentation

**DOI:** 10.1186/s12911-023-02189-1

**Published:** 2023-05-10

**Authors:** Peng Sun, Zengnan Mo, Fangrong Hu, Xin Song, Taiping Mo, Bonan Yu, Yewei Zhang, Zhencheng Chen

**Affiliations:** 1 School of Electronic Engineering and Automation, Guilin University of Electronic Technology, Guilin, 541004 Guangxi China; 2 Center for Genomic and Personalized Medicine, Guangxi Medical University, Nanning, 530021 Guangxi China; 3 School of Architecture and Transportation Engineering, Guilin University of Electronic Technology, Guilin, 541004 Guangxi China; 4 Hepatopancreatobiliary Center, The Second Affiliated Hospital of Nanjing Medical University, Nanjing, China

**Keywords:** Medical image segmentation, Kidney tumor segmentation, 2.5D model, MFFAU-Net, KiTS19, KiTS21

## Abstract

**Background:**

Kidney tumors have become increasingly prevalent among adults and are now considered one of the most common types of tumors. Accurate segmentation of kidney tumors can help physicians assess tumor complexity and aggressiveness before surgery. However, segmenting kidney tumors manually can be difficult because of their heterogeneity.

**Methods:**

This paper proposes a 2.5D MFFAU-Net (multi-level Feature Fusion Attention U-Net) to segment kidneys, tumors and cysts. First, we propose a 2.5D model for learning to combine and represent a given slice in 2D slices, thereby introducing 3D information to balance memory consumption and model complexity. Then, we propose a ResConv architecture in MFFAU-Net and use the high-level and low-level feature in the model. Finally, we use multi-level information to analyze the spatial features between slices to segment kidneys and tumors.

**Results:**

The 2.5D MFFAU-Net was evaluated on KiTS19 and KiTS21 kidney datasets and demonstrated an average dice score of 0.924 and 0.875, respectively, and an average Surface dice (SD) score of 0.794 in KiTS21.

**Conclusion:**

The 2.5D MFFAU-Net model can effectively segment kidney tumors, and the results are comparable to those obtained with high-performance 3D CNN models, and have the potential to serve as a point of reference in clinical practice.

## Introduction

Nowadays, cancer tumors are increasingly threatening human health; tumors of the kidney are among the most frequently occurring tumors in the urinary system [1]. Most Kidney tumors are malignant, and their incidence is second only to bladder cancer [2]. Clinically common kidney tumors include kidney carcinoma derived from the kidney parenchyma, Wilms tumor, and transitional cell papilloma arising in the kidney pelvis and calyx [3]. Among adult malignant tumors, kidney cell carcinomas (RCC) account for all 80-85% of primary kidney tumors. Next is transitional cell carcinoma of the kidney pelvis, which accounts for about 8%. In other kidneys, parenchymal epithelial tumors are less common. In addition, such as kidney adenoma, kidney cyst, kidney hemangioma, kidney hamartoma, kidney lipoma, etc., are benign tumors [4–7]. So far, kidney cancer’s etiology is unclear, and it may be related to many factors, such as environmental factors, genetic factors, and so on [8, 9]. Most people have kidney tumors without feeling anything; sometimes, they are found in the advanced stages of cancer. Doctors can use medical imaging techniques such as CT, MRI, and ultrasound to visualize the kidneys and tumors, and analyze their size, shape, and appearance. This information can be utilized to determine the best possible treatment options for patients [10, 11]. Unfortunately, identifying benign and malignant tumors directly from large numbers of patient kidney images is challenging. In addition, manual segmentation of kidney tumors significantly consumes the doctor’s energy and time and wastes medical resources [12]. In addition, manual segmentation usually depends on the experience of doctors. For example, when an inexperienced doctor finds the tumor inert after nephrectomy, the patient’s surgical treatment will be regarded as over-treatment because the patient has lost kidney function.

CNNs have led to rapid development in the field of image segmentation, particularly in medical image segmentation [13, 14]. CNN can automatically complete kidney segmentation without human intervention. Pandey et al. [15] analyzed multiple segmentation methods in kidney images. Their analysis accuracy, dataset size, and the impact of pathological kidneys on segmentation performance. Furthermore, they discussed the challenges associated with kidney segmentation and assessed the performance of these segmentation methods. The KiTS19 Challenge dataset includes 210 CT scans of patients’ kidneys and the corresponding label contained two parts: kidney and tumor, and did not contain some benign tumors, such as cysts. Therefore, the KiTS19 challengers strived to enhance the accuracy of tumor segmentation. In 2021, KiTS21 updates the KiTS19 dataset, releasing 300 patient kidney images, and adding cysts to the label. The participating teams were tasked with creating the most effective method for segmentation of tumor. In the study of renal tumor segmentation, most participants of KiTS19 and KiTS21 used neural network-based cascade segmentation structure. Some experts also proposed the method to remove sections irrelevant to the research interest from the kidney volume medical image [16]. Still, none of the methods has been evaluated as the best CNN method. Therefore, continued research in the renal tumor segmentation area remains significant, particularly in the case of kidney and tumor segmentation where cysts cyst segmentation cannot be overlooked.

Currently, 2D CNNs are used more in medical image processing due to the lower memory requirements and faster training time, However, 2D CNNs do not make use of 3D features, which can limit their performance in certain tasks. Therefore, in 3D kidney volume segmentation, it is difficult for 2D segmentation to acquire inter-slice features [17]. On the other hand, the popularity of three-dimensional (3D) CNNs for medical image segmentation has increased significantly, owing to their ability to learn hierarchical features across slices. In contrast to 2D CNNs, 3D CNNs requires more GPU memory consumption and significant computational costs. Additionally, these 3D models necessitate extensive fully 3D annotated datasets, further contributing to their limitations. The primary focus of this paper is the limitation of existing 2D models to acquire inter-slice features and the excessive computational resource requirements of 3D networks. We propose a trainable 2.5D MFFAU-Net model that balances memory consumption and model complexity. This paper offers several significant contributions, including:

1. This paper proposes a 2.5D scheme for kidney segmentation. This scheme inputs multiple slices in the input layer of the model, and the slices in the middle correspond to them in the output layer. By using this 2.5D scheme, the model can obtain 3D information about the slices by performing 2D convolutions on the multiple slice inputs and intermediate slice outputs.
2. In this paper, the MFFAU-Net model is proposed for kidney tumor segmentation. In the encoding and decoding stages, we use the ResConv architecture and analyze the spatial information between slices using multi-level features. These features are essential for accurate segmentation and help the model improve the segmentation results.
3. We evaluated the model proposed in this paper on both KiTS19 and KiTS21 kidney CT datasets, and both obtained good segmentation results.

This paper has adopted the following structure: Sect. 2 presents a review of related research work, Sect. 3 provides the proposed model’s design, Sect. 4 reports the experimental results obtained through the model’s implementation, and Sect. 5 compares and discusses the proposed method with other state-of-the-art methods. Section 6 concludes the findings and contributions.

## Related work

Despite the many traditional CT image segmentation techniques proposed in the past few decades, such as manual, threshold, atlas, graph, and hybrid segmentation, they have limitations in accurately segmenting kidneys on CT images. For instance, simple procedures like threshold segmentation are sensitive to noise and cannot handle significant intensity variations in CT; It is worth noting that atlas and threshold segmentation is not automatic, and their segmentation performance can be affected by inter-rater variability.

Ronneberger et al. [18] used the U-Net model to complete the task of medical image segmentation at ISBI in 2015. However, he only used 30 images and the data expansion strategy to achieve a meager error rate and won the ISBI championship. Subsequently, variant U-Net based algorithms were applied in many fields of image processing and achieved good results.

In 2021, Heller et al. [19] summarized the top five methods results of the KiTS19 challenge. In their paper, we have found that the segmentation models of the top five contestants are all related to U-Net, and the 1st was made by Fabian et al. [20]. Three 3D U-Net architectures were tested and the submission scored a dice of 0.974 and 0.851 for kidney and tumor resulting in 0.912 composite scores. Therefore, the authors claimed that the power of the 3D U-Net method is sufficient for the best results. Some researchers who failed to participate in the KiTS19 challenge in time also proposed various kidney and tumor segmentation schemes. Kang et al. [21] introduced an a priori contour auxiliary channel in a 2D network to segment ROI regions containing kidneys and kidney tumors, used it as the input for subsequent fine segmentation, and then used ConvLSTM to extract spatial correlation slices and combined with 3D CNN for fine segmentation. Eventually, they achieved 0.964 kidney dice and 0.789 tumor dice. da Cruz et al. [22] used AlexNet to narrow down the problem, used image processing techniques to reduce false positives, and then used 2D U-Net to complete kidney segmentation. Finally, they achieved 0.930 kidney dice. Pandey [23] used prior knowledge of the shape, size, and position of the kidney relative to the spine to locate kidney ROI. Then they used a 3D U-Net to perform left and right kidney segmentation, respectively. Finally, they tested the method on CT data from 21 patients with KiTS19 and obtained a kidney dice score of 0.976.

KITS21 announced the kidney tumor segmentation models of some contestants. Shen et al. [24] used the COTRNet model for kidney segmentation. To learn multi-scale features, the designed encoder consists of ResNet, Transformer encoder layer, Pretrained model, and Deep supervision. Finally, their method ranked 22 and achieved 0.923 kidney dice, 0.553 mass dice, and 0.506 tumor dice. Adam et al. [25] used a 3D U-ResNet method for kidney segmentation, they introduced the Residual Block in the U-Net model and used rule-based post-processing to eliminate false positive artifacts. Ultimately, they led to the 12th position in the KiTS21. Zhao et al. [26] used the nnU-Net-based coarse-to-fine framework to get 1st in the KiTS21, and they performed dice scores of 0.975, 0.885, 0.869 for kidney, mass, tumor, respectively.

In conclusion, despite the use of various approaches as kidney segmentation models, the majority of kidney tumor segmentation studies continue to rely on the cascaded architecture as the primary model. However, 3D models will require more computational resources, and 2D models cannot acquire spatial information. Therefore, this paper proposes a novel segmentation approach for kidneys and tumors, aiming to address the computational complexity issue associated with 3D CNNs while ensuring adequate segmentation accuracy. The objective is to enhance the neural network architecture without compromising the accuracy and present a universal methodology applicable to kidney tumor segmentation. We not only focus on the segmentation results of the model in KiTS19 or KiTS21 but also focus on the segmentation results in KiTS19 and KiTS21 to aid physicians in the rapid diagnosis of patients.

## Methodology

In kidney tumor segmentation, most research still rely on the 3D CNN architecture as their fundamental model. The 3D convolution has the problems of high computational cost and large memory consumption of GPU, which cannot quickly complete the segmentation task. Thus, to rapidly segment the tumor from the kidney image, the 2D image segmentation algorithm is a suitable solution. When it comes to segmenting the kidney and tumor from a sequence of CT image slices, a 2D scheme is often used where the 3D image is converted into 2D slices and processed using 2D convolutions. However, this approach does not exploit the spatial structure in the 3D image, which make segmentation more challenging. Therefore, this paper proposes a cascaded 2.5D MFFAU-Net model that balances accuracy and computational resources for the kidney segmentation task, capable of incorporating 3D information introduced into CT images using 2D convolutions.

**Fig. 1:**
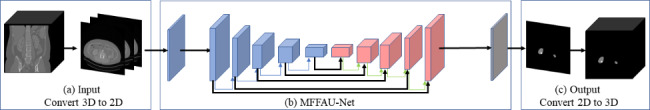
2.5D MFFAU-Net model architecture. **(a)** Input, **(b)** MFFAU-Net model, **(c)** Output

The 2.5D model is illustrated in Fig. 1. Part (a) is the input, part (b) is the MFFAU-Net model, and part (c) is the output. Parts (a) and (c) construct a 2.5D convolution strategy. First, the original 3D volume image is transformed into several consecutive 2D slices in part (a). Then, a stack of multiple adjacent slices is used (3 × 512 × 512 in this paper) as the input image for the model. Next, in part (c), the segmentation result (1 × 512 × 512 in this paper) corresponding to the middle CT image is used as the output image of the model. Finally, consecutive 2D segmentation results are converted into 3D images to obtain the completed 3D segmentation results. By using a multi-slice input, the model can incorporate more image content from the axial plane. Therefore, in the 2.5D model, it is possible to introduce some of the 3D information by only using 2D convolutions.

**Fig. 2:**
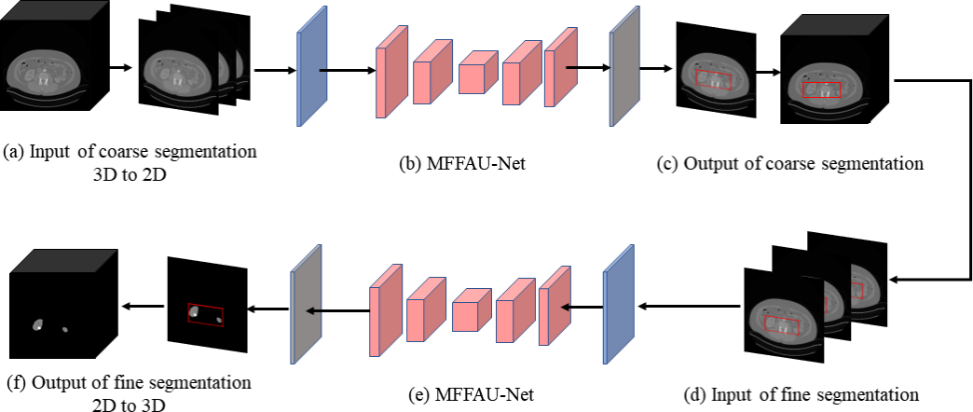
The cascade segmentation model of kidney tumors

Figure 2 shows the cascade segmentation model of kidney tumors in this paper. The cascading model is composed of two 2.5D MFFAU-Net model architectures. Firstly, **(a)** - **(c)** constitute a coarse segmentation framework and divide the kidney ROI. **(d)** - **(f)** constitutes the fine segmentation framework, with the input being the kidney ROI results of coarse segmentation. In KiTS19, the output of fine segmentation is the kidney tumor. In KiTS21, the outputs are masses, tumors, and cysts. Cascade segmentation can eliminate the interference of background information and narrow the range of tumors and cysts that need to be segmented by roughly segmenting the selected kidney ROI. The kidney ROI will provide smaller dimensions and more accurate information for subsequent segmentation.

**Fig. 3:**
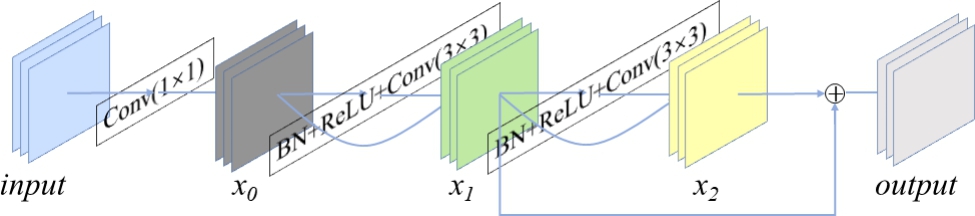
ResConv architecture’s composition. 1 × 1: kernel size = 1 × 1; BN: batch normalization; ReLU: activation function; 3 × 3: kernel size = 3 × 3

In the model’s encoding and decoding paths, we propose using the ResConv architecture to replace the original 3 × 3 convolution. The ResConv architecture’s composition is shown in Fig. 3.

ResConv consists of a 1 × 1 convolution and two residual blocks. First, in Eq. (1), we change the number of channels of feature maps of the original input image using a 1 × 1 convolution.

1 $${x}_{0}=Conv(1\times 1)input$$

Then, Eqs. (2) and (3) compute the results of the two residual blocks.

2 $${x}_{1}=\left(Conv\left(3\times 3\right)+BN+Relu\right){x}_{0}+{x}_{0}$$

3 $${x}_{2}=\left(Conv\left(3\times 3\right)+BN+Relu\right){x}_{1}+{x}_{1}$$

Finally, Eq. (4) adds the results of the two residual blocks and shows the final output.

4 $$output={x}_{1}+{x}_{2}$$

ResConv contains the results of two residual blocks so that the ResConv can capture more information.

**Fig. 4:**
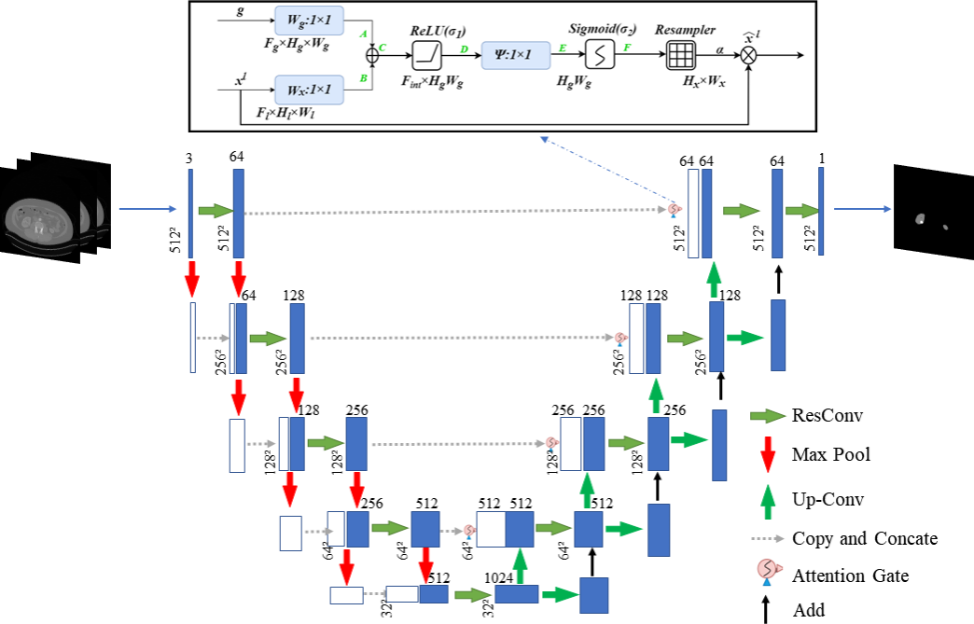
The specific architecture of the MFFAU-Net model

The MFFAU-Net model is a modified version using the standard U-Net, and the architecture’s composition is illustrated in Fig. 4. The MFFAU-Net model shows two encoding paths. To connect the input and output, we utilize the ResConv architecture and Max Pooling in the first encoding path. In the second path, we only use Max Pooling without ResConv to down-sample the feature maps and concatenate them with the input, reducing the input by half. Then, use Skip Connections to connect outputs of the two encoding paths to form the input of the next convolution operation, which enables the model to learn feature information at different levels better. There are also two paths in the decoding part. The first decoding path is symmetric with the encoding path, but Max Pooling is replaced by up-sampling. To establish communication between the encoding and decoding paths at the same level, we employed Skip Connections in our architecture. Then go through the ResConv layer to get the output of the first decoding path. In addition, the second path of the decoding layer doubles the input using Up-sampling. It then concatenates with the result of the first decoding path to get the final outcome of the layer.

Because there is a lot of feature information in the coding path. Still, much of them are unnecessary and redundant data. The attention gate is equivalent to filtering the current layer of the encoder to suppress irrelevant information in the image and highlight essential local features, thus improving the robustness of the model. To enhance the performance of the MFFAU-Net, the model incorporated an attention gate into the Skip Connection section, as shown in the top half of Fig. 4.

1. The encoder output x^l^ and the decoder output g are the two inputs of the attention gate. A and B are the results of the one-dimensional operation on g and x^l^ simultaneously. A and B are two feature maps of the same size and channel number, but the features extracted by A and B differ. So then, C = A + B, C can strengthen the same region of interest signal compared with A and B.
2. We used the Relu activation function and 1 × 1 convolution to convert C into D and E.
3. Then, we used the sigmoid activation function to get F.
4. F Restored the size through a Resampler module to get the weight information α.
5. Finally, we used the weight information α multiplied by the original input x^l^ to obtain the activation function ${\widehat{x}}^{l}$ of the attachment of x^l^. Next, the feature maps are weighted using attention coefficients. These coefficients are generated by calculating the similarity between the outputs of the current encoder layer and the next decoder layer.

## Experimental datasets and results

This section evaluated the MFFAU-Net method. First, we introduced the kidney datasets used for the experiments, then presented the proposed method’s experimental results in detail.

### Dataset description

This paper aims to build a general system suitable for kidney tumor segmentation. Therefore, we used two datasets, KiTS19 and KiTS21.

The first dataset used in this study is KiTS19, KiTS19 is a public data set, and the official website of KiTS19 contains relevant descriptions of patient data (https://kits19.grand-challenge.org). Therefore, we can use it directly without obtaining the patient’s informed consent again. The KiTS19 dataset excluded patients with cysts and tumor thrombi since it was challenging to identify the boundaries of tumors beyond the primary site. CT images and labels of patients are provided in anonymous NIFTI format with the shape num_slices-height-width. During image acquisition for the KiTS19 dataset, the patients were in a supine position, resulting in the origin of the height-width being at the left front of the patient. The num_slices in the KiTS19 dataset correspond to the axial view of the kidney, and as the slice index increases from top to bottom, the view progresses through the kidney. In addition, 210 scans and labels are included in the KiTS19 dataset, which we can download from https://github.com/neheller/kits19. To evaluate the segmentation performance in KiTS19, the category of kidney includes both kidneys and tumors, while the category of tumor only considers tumors as the foreground.

The second dataset used in this paper is KiTS21, which was collected from patients who underwent partial or radical nephrectomy to treat suspected kidney malignancy at M Health Fairview or Cleveland Clinic Medical Center over a period spanning from 2010 to 2020. These cases were reviewed retrospectively to identify all patients who underwent patients with contrast-enhanced preoperative CT scans of kidneys. KiTS21 is a public data set, and the official website of KiTS21 contains relevant descriptions of patient data (https://kits21.kits-challenge.org). Therefore, we can use it directly without obtaining the patient’s informed consent again. The definition of kidney and tumor in KiTS21 was the same as that of kidney and tumor in KiTS19 and added cysts. 300 scans with high-quality ground truth segmentation are included in the KiTS21 dataset, which we can download from https://github.com/neheller/kits21. KITS21 proposed to use the HECs to obtain relatively comprehensive metrics. The kidney HEC considers the kidney, tumor, and cyst as foreground classes in KITS21; the kidney mass HEC containing tumor and cysts; and the tumor HEC only considers the tumor as the foreground class. However, cyst segmentation should not be ignored, and we propose a cyst definition: the cyst category considers the cyst as the foreground only to determine the effectiveness of cyst segmentation.

### Experimental results

Both KiTS19 and KiTS21 used the dice similarity coefficients to evaluate the performance of the models. The dice is commonly used in image processing to analyze the performance of segmentation methods on ground truth [27]. The calculation method for the dice similarity coefficient is shown in Eq. (5).

5 $$\stackrel{-}{Dice}=\frac{1}{n}\sum _{1}^{n}\frac{2\left|{A}_{i}\cap {B}_{i}\right|+1}{\left|{A}_{i}\right|+\left|{B}_{i}\right|+1} i=1, 2, \dots ,n.$$

In Eq. (5), *A*_*i*_ denotes the label of the i-th image, and *B*_*i*_ denotes the corresponding segmentation result.

KiTS21 uses Surface dice (SD) as an evaluation index for kidney tumor segmentation. Therefore, we also calculated SD in the test of KiTS21.

This paper used the 2.5D MFFAU-Net model to segment kidney tumors. First, in order to reduce the effects of non-organic artifacts, we pre-process the dataset. Next, we uniformly use the 512 × 512 pixels for model input. Subsequently, used the mean and standard deviation of the normal foreground to enhance the network’s training performance. Finally, we use the cascaded model to segment kidney ROI, mass and tumor.

We tested the segmentation effect of 2.5D MFFAU-Net in the KITS19 and KiTS21 datasets. All the data were introduced in Sect. 4.1, and no other datasets have been used. We randomly selected 20% of patients’ CT data for testing, 42 CT scans for KiTS19 and 60 for KiTS21. Then, 80% for training. At the same time, we have done ablation research and tested the segmentation effects of U-Net, ResU-Net, DenseU-Net, and V-Net, respectively. All models used the same training and test sets for each test, and were trained on three computers with NVIDIA GeForce RTX 3090 Ti (24GB) graphics processing unit (GPU). We trained the model for a total of 100 epochs and the training process will automatically stop when the model has no performance improvement for 30 consecutive epochs. During the test, the MFFAU-Net needs about 7 s to obtain the segmentation results of a patient in KiTS19 in KiTS21.

Table 1 shows the segmentation results in KiTS19. There are two types of input: 3 × 512 × 512 and 5 × 512 × 512. In the segmentation results with 3 adjacent slices as input, the MFFAU-Net (ResConv) used longer training time and achieved 0.975 kidney dice, outperforming U-Net (0.950), ResU-Net (0.961), DenseU-Net (0.963), V-Net (0.965) and MFFAU-Net with original convolution (0.972). Furthermore, MFFAU-Net achieved 0.872 tumor dice, outperforming U-Net (0.779), ResU-Net (0.856), DenseU-Net (0.852), V-Net (0.850) and MFFAU-Net with original convolution (0.868). In addition, the segmentation results with 5 adjacent slices as input show that more slices would benefit from a wider context on the z-axis in U-Net, ResU-Net, and V-Net. But it brought worse results on DenseU-Net and MFFAU-Net. Table 1 can show that the 2.5D MFFAU-Net (ResConv) with 3 adjacent slices as input had good segmentation performance in KiTS19.

**Table 1:** Segmentation results in KiTS19

Algorithm or method	Input	Training time	Kidney Dice	Tumor Dice
2.5D U-Net	3 × 512 × 512	76 h	0.950	0.779
	5 × 512 × 512	87 h	0.950	0.788
2.5D ResU-Net	3 × 512 × 512	82 h	0.961	0.856
	5 × 512 × 512	94 h	0.965	0.855
2.5D DenseU-Net	3 × 512 × 512	83 h	0.963	0.852
	5 × 512 × 512	96 h	0.962	0.850
2.5D V-Net	3 × 512 × 512	96 h	0.965	0.850
	5 × 512 × 512	103 h	0.970	0.855
2.5D MFFAU-Net (original conv)	3 × 512 × 512	92 h	0.972	0.868
	5 × 512 × 512	105 h	0.972	0.865
2.5D MFFAU-Net (ResConv)	3 × 512 × 512	104 h	0.975	0.872
	5 × 512 × 512	133 h	0.970	0.860

Table 2 shows the dice and SD results in KiTS21. There are two types of input: 3 × 512 × 512 and 5 × 512 × 512. In the dice results with 3 adjacent slices as input, the MFFAU-Net needs 129 h of training and achieved 0.973 kidney dice, outperforming U-Net (0.949), ResU-Net (0.953), DenseU-Net (0.961), V-Net (0.967) and MFFAU-Net with original convolution (0.968). Furthermore, MFFAU-Net achieved 0.887 mass dice, outperforming U-Net (0.808), ResU-Net (0.815), DenseU-Net (0.811), V-Net (0.853), and MFFAU-Net with original convolution (0.877). In addition, MFFAU-Net achieved 0.873 tumor dice, outperforming U-Net (0.702), ResU-Net (0.779), DenseU-Net (0.753), V-Net (0.798) and MFFAU-Net with original convolution (0.795). Finally, MFFAU-Net achieved 0.768 cyst dice, outperforming U-Net (0.580), ResU-Net (0.622), DenseU-Net (0.591), V-Net (0.653), and MFFAU-Net with original convolution (0.651). In the SD results with 3 adjacent slices as input, The MFFAU-Net (ResConv) achieved 0.941 kidney SD, 0.788 mass SD, 0.769 tumor SD, and 0.678 cyst SD. The MFFAU-Net (ResConv) got the excellent segmentation performance in dice and SD. In addition, we also tested the segmentation results with 5 adjacent slices as input in KiTS21. Unfortunately, the result is not better than MFFAU-Net with 3 adjacent slices as input. Table 2 shows that the ResU-Net model and DenseU-Net model have similar segmentation performance and prove that MFFAN-Net has good segmentation performance in KiTS21.

**Table 2:** Dice and SD results in KiTS21

Algorithm or method	Input	Training time	Kidney Dice/SD	Mass Dice/SD	Tumor Dice/SD	Cyst Dice/SD
2.5D U-Net	3 × 512 × 512	90 h	0.949/0.912	0.795/0.663	0.702/0.581	0.580/0.452
	5 × 512 × 512	106 h	0.951/0.915	0.795/0.662	0.700/0.578	0.585/0.460
2.5D ResU-Net	3 × 512 × 512	93 h	0.953/0.914	0.815/0.688	0.779/0.669	0.622/0.522
	5 × 512 × 512	110 h	0.956/0.915	0.818/0.700	0.780/0.672	0.620/0.515
2.5D DenseU-Net	3 × 512 × 512	96 h	0.961/0.930	0.811/0.683	0.753/0.648	0.591/0.500
	5 × 512 × 512	114 h	0.961/0.932	0.815/0.686	0.750/0.650	0.600/0.505
2.5D V-Net	3 × 512 × 512	112 h	0.967/0.934	0.853/0.715	0.798/0.698	0.653/0.541
	5 × 512 × 512	122 h	0.965/0.930	0.854/0.718	0.801/0.700	0.658/0.550
2.5D MFFAU-Net (original conv)	3 × 512 × 512	116 h	0.968/0.935	0.877/0.746	0.795/0.688	0.651/0.551
	5 × 512 × 512	137 h	0.965/0.932	0.875/0.742	0.800/0.696	0.648/0.543
2.5D MFFAU-Net (ResConv)	3 × 512 × 512	129 h	0.973/0.941	0.887/0.788	0.873/0.769	0.765/0.678
	5 × 512 × 512	153 h	0.972/0.938	0.886/0.788	0.858/0.748	0.729/0.622

**Fig. 5:**
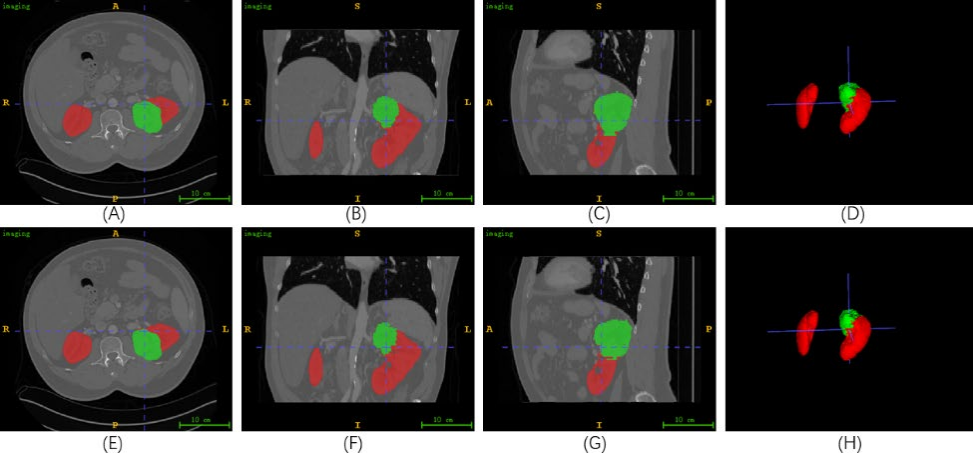
The results of case 149 in KiTS19. The ITK-SNAP software is used to visualize the segmentation results. **(A)**-**(C)** display the label of the 61st slice in the axial plane, the 328th slice in the sagittal plane, and the 261st slice in the coronal plane. **(D)** illustrates the ground truth of the 3D view. On the other hand, Figures **(E)**-**(H)** exhibit the corresponding predicted values. In these figures, kidney is highlighted in red, while tumor is highlighted in green

The results of case 149 in KiTS19 are presented in Fig. 5. (A)-(D) depict the label of the axial, sagittal, coronal, and 3D views, respectively. On the other hand, (E)-(H) demonstrate the predicted values. KiTS19 only divided the whole kidney into two categories: kidney and tumor, so only two colors are shown in the image. The color red is used to represent the kidney, while green is used to represent the tumor in the segmentation results. To better visualize the 3D structure of the kidney, we rotated the 3D view. (E)-(G) demonstrated that the 2.5D MFFAU-Net can segment kidney and tumor regions from each slice in multiple planes. (H) demonstrates that the 2.5D MFFAU-Net is capable of accurately segmenting the precise locations of kidneys and tumors, thereby indicating its potential to be used in clinical practice.

**Fig. 6:**
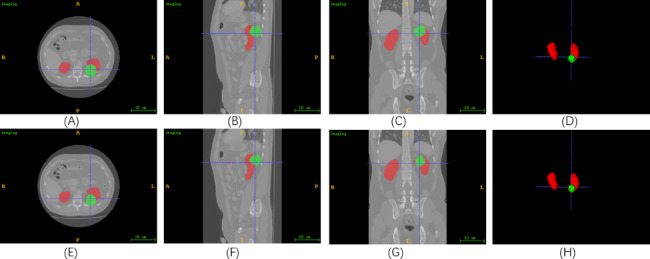
The results of case 131 in KiTS19. The ITK-SNAP software is used to visualize the segmentation results. **(A)**-**(C)** display the label of the 27th slice in axial plane, the 329th in sagittal plane, and the 335st in coronal plane. D) illustrates the ground truth of the 3D view. On the other hand, Figures **(E)**-**(H)** exhibit the corresponding predicted values

The results of case 131 in KiTS19 are presented in Fig. 6. (E) - (G) show that 2.5D MFFAU-Net has a slight deviation in kidney segmentation, which corresponds to the reason for low kidney dice in Table 1. (H) demonstrates that 2.5D MFFAU-Net can segment specific locations of kidneys and tumors in KiTS19. Overall, 2.5D MFFAU-Net can effectively segment kidneys and tumors in KiTS19.

**Fig. 7:**
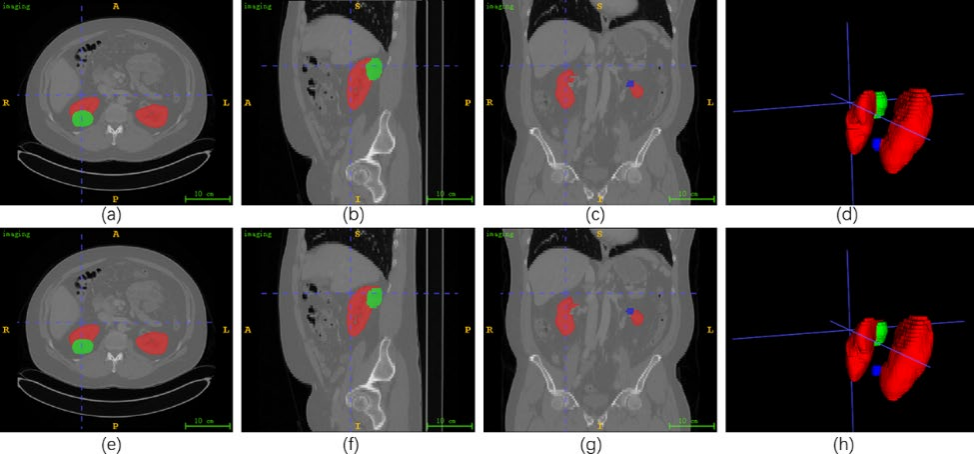
The results of case 243 in KiTS21. The ITK-SNAP software is used to visualize the segmentation results. **(a)**-**(c)** display the label of the 30th slice in axial plane, the 167th in sagittal plane, and the 237th in coronal plane. **(d)** show the ground truth of 3D views. **(e)**-**(h)** exhibit the corresponding predicted values. In these figures, kidney is labeled in red, tumor in green, and cyst in blue

Figure 7 shows the results of case 243 in KiTS21. **(a)**-**(d)** depict the label of the axial, sagittal, coronal, and 3D views, respectively. On the other hand, **(e)**-**(h)** demonstrate the predicted values. Different from KiTS19, in the KiTS21 dataset, the entire kidney is classified into three categories: kidney, tumor, and cyst, kidney is labeled in red, tumor in green, and cyst in blue. In order to better observe the kidney 3D structure, we rotated the 3D view. We can find from **(h)** that the structure of the cyst in case 243 is small and easily overlooked, and the tumor and cyst are not on the same kidney. This case requires evaluating both the tumor and cyst to formulate a better treatment plan. **(e)**-**(h)** prove that the 2.5D MFFAU-Net can not only segment the specific locations of kidneys tumors in KiTS21 but also maintain an excellent segmentation effect for smaller objects (such as cysts).

**Fig. 8:**
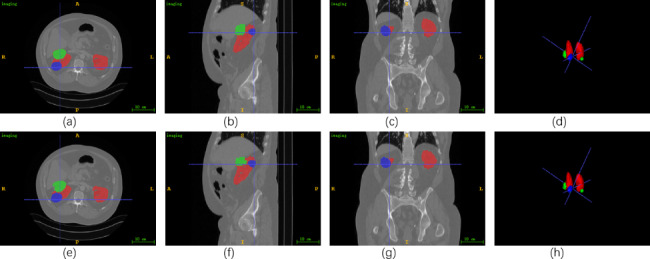
The results of case 58 in KiTS21. The ITK-SNAP software is used to visualize the segmentation results. **(a)**-**(c)** display the label of the 30th slice in axial plane, the 171st in sagittal plane, and the 309th in coronal plane. **(d)** show the ground truth of 3D views. **(e)**-**(h)** exhibit the corresponding predicted values

Figure 8 shows the results of case 58 in KiTS21. (a)-(d) depict the label of the axial, sagittal, coronal, and 3D views, respectively. On the other hand, (e)-(h) demonstrate the predicted values. In order to better observe the kidney 3D structure, we rotated the 3D view. We can find from (d) and (h) that the structure of the cyst in Case 58 is minimal and easily overlooked, and every kidney contains a tumor, which is a severe kidney tumor patient. In addition, the left kidney has both kidneys and cysts, which is a complex situation. Considering the preservation of kidney function, more accurate segmentation results are needed to help formulate better treatment plans. (e) - (h) demonstrated that the 2.5D MFFAU-Net could segment specific kidney and tumor locations in 3D CT images and maintain good segmentation results for smaller objects such as cysts. 2.5DMFFAU-Net can effectively segment kidneys, tumors, and cysts in KiTS21.

Tables 1 and 2; Figs. 5, 6, 7 and 8 demonstrated that the 2.5D MFFAU-Net has good segmentation performance in kidney, tumor, and cyst segmentation, especially the MFFAU-Net has been shown to effectively capture minor features such as tumors and cysts. In addition, segmentation of minor features can assess the aggressiveness and complexity of tumors, which can help physicians plan diagnosis and treatment and avoid overtreatment of kidney tumors. Therefore, we believe that the 2.5D MFFAU-Net can serve as a reference model for kidney tumor segmentation.

## Discussion

This paper presented a 2.5D cascade model with multi-level feature fusion for kidney tumor segmentation. The results of the MFFAU-Net in both KiTS19 and KiTS21 datasets have been demonstrated to be good through Tables 1 and 2; Figs. 5 and 6. Furthermore, we covered the state-of-the-art methods. In Table 3, we can see a comparison with state-of-the-art approaches in terms of performance for kidney tumor segmentation in KiTS19. As the training and testing divisions vary between the studies that employed these methods, it should be emphasized that each study employed various validation techniques. Therefore, we utilized their results directly.

**Table 3:** Comparison with the state-of-the-art methods in KiTS19.

Authors	methods	Kidney Dice	Tumor Dice
This paper	2.5D MFFAU-Net	0.975	0.872
Fabian et al. [20].	3D U-Net	0.974	0.851
Kang et al. [21].	3D-CNN + ConvLSTM	0.964	0.789
da Cruz et al. [22].	AlexNet + 2D U-Net	0.930	\
Pandey et al. [23].	active contour + deep learning	0.976	\
da Cruz et al. [28].	2.5D DeepLabv3+	\	0.852
Causey et al. [29].	Ensemble of U-Net	0.949	0.601
Kittipongdaja et al. [30].	2.5D DenseU-Net	0.960	\
Türk et al. [31].	Hybrid V-Net	0.977	0.865
Türk et al. [32].	Two-Stage Bottleneck Block	\	0.869

Fabian et al. [20] segmented kidneys and tumors using 3D U-Net architectures. The authors stated that the 3D U-Net has great potential for achieving excellent results. They were able to achieved the highest performance in the KiTS19 challenge with 0.974 kidney dice and 0.857 tumor dice, resulting in a composite score of 0.912. Compared with 3D-U-Net, the 2.5D structure we use can save more training time, and the results from the experiments presented in this paper suggest that MFFAU-Net outperforms 3D U-Net in segmentation tasks.

Kang et al. [21] used a method that combined ConvLSTM for spatial information extraction between slices and 3D-CNN for kidney segmentation. The authors proposed that the ConvLSTM module could be utilized in the down-sampling process to capture the strong correlation between the 3D CT slices. They randomly selected 20 cases as the validation set and obtained average scores of 0.964 and 0.789 for kidneys and tumors. Our model significantly improved kidney and tumor segmentation compared to their model.

da Cruz et al. [22] proposed a modified version of the 2D U-Net, incorporating AlexNet for feature extraction, to improve the kidney and tumor segmentation. They reported achieving a 0.930 kidney Dice with this approach. Unfortunately, their study did not acquire 3D information on the kidneys and did not study the segmentation of the tumor, which prevented doctors from planning a diagnosis of the tumor in subsequent surgical protocols.

Pandey et al. [23] developed a method based on active contour and deep learning techniques. First, the author used prior knowledge information of the kidney relative to the spine to locate the kidney region and cut the left and right kidneys into two pieces with a size of 128 × 128 × 128 as an independent image of the kidney region. Then they used the 3DU-Net model to perform left and right kidney segmentation, respectively. Finally, they combined the segmentation results of the two kidneys to obtain the final kidney segmentation image. They were evaluated on CT data from 21 patients with KiTS19, and the results showed a kidney dice score of 0.976. Single kidney segmentation treats tumors as kidneys without interference from tumor types. Therefore, using prior knowledge or image processing methods can achieve high kidney scores. However, tumor segmentation is also essential, as accurate segmentation can help clinicians formulate surgical plans.

da Cruz et al. [28] employed the DeepLabv3 + 2.5D model to perform tumor segmentation. In their research, they removed the fully connected layer from the DPN-131 and used it as the encoder in the DeepLabv3 + model. The authors claimed that DPN had the advantages of ResNet model and DenseNet model. In their study, da Cruz et al. randomly partitioned the provided dataset of 210 CTs from the KiTS19 challenge into training, validation, and testing sets consisting of approximately 147, 32, and 31 volumes, respectively. They achieved a tumor score of 0.852 without achieving kidney segmentation, and their results were similar to those of our tested 2.5D ResU-Net and 2.5D DenseU-Net in Table 1. Compared to their model, we achieved kidney and tumor segmentation in KiTS19.

After testing many models, an ensemble of U-Net deep learning models was proposed by Causey et al. [29]. They named the modified models the KT model in the coarse segmentation and the KT/T in fine segmentation. In their study, they used 168 scans with ground-truth segmentation for training their ensemble of U-Net deep learning models, and achieved 0.949 kidney dice and 0.601 tumor dice.

Kittipongdaja et al. [30] used 2.5D ResU-Net and 2.5D DenseU-Net to achieve kidney segmentation, and they did not study tumor segmentation. They achieved 0.960 kidney dice using 2.5D DenseU-Net in KiTS19. The outcomes they achieved were comparable to those obtained using the 2.5D DenseU-Net model we tested in Table 1. The 2.5D MFFAU-Net model outperformed the models they tested on kidney segmentation.

Türk et al. [31] developed a hybrid V-Net that utilized the strengths of V-Net models to improve the encoder and decoder levels, resulting in a more successful segmentation system. The decoder module was designed of the ET-Net structure, and the encoder module captured all features during the segmentation process through an integrated V-Net model. As a result, they achieved high average dice scores of 0.977 and 0.865 for kidney and tumor. The MFFAU-Net had a lower kidney dice but achieved better tumor dice, possibly because the hybrid V-Net using 3D images had more contextual information and was more sensitive to the extensive features (kidneys). On the other hand, the 2.5D MFFAU-Net using ResConv convolution structure and attention gate mechanism paid more attention to minor features (tumors).

Türk et al. [32] introduced a new architecture for kidney tumor segmentation that consisted of three stages. the encoder was modified to include SE blocks for better learning of image features in the first stage. The second stage used a decoder based on the ResNet + + structure to capture finer details before the output layer. The third stage integrated the NLB + GAB structure to address the bottleneck issue commonly encountered in convolutional neural networks. Finally, 0.869 tumor dice were obtained in a test set of 20 kidney data.

Table 4 presents a comparison with other state-of-the-art methods in KiTS21. As the training and testing divisions vary between the studies that employed these methods, it should be emphasized that each study employed various validation techniques. Therefore, we utilized their results directly.

**Table 4:** Comparison with the state-of-the-art methods in KiTS21.

Authors	methods	Kidney Dice/SD	Mass Dice/SD	Tumor Dice/SD	Cyst Dice/SD
This paper	2.5D MFFAU-Net	0.973/0.941	0.887/0.788	0.873/0.769	0.765/0.678
Shen et al. [24].	COTRNet	0.923/0.885	0.553/0.369	0.506/0.355	\
Adam et al. [25].	3D U-ResNet	0.951/0.904	0.798/0.648	0.781/0.627	\
Zhao et al. [26].	nnU-Net	0.975/0.949	0.885/0.787	0.869/0.769	\
Zang et al. [33].	3D U-Net	0.970/0.937	0.851/0.720	0.819/0.700	\
Chen et al. [34].	ResSENormUnet + DenseTransUnet	0.943/ \	\	0.778/ \	0.710/ \
Li et al. [35].	3D deep learning	0.960/ \	\	0.815/ \	0.450/ \
George [36].	3D U-Net	0.975/0.945	\	0.871/0.761	0.881/0.773

Shen et al. [24] presented the COTRNet model for kidney tumor segmentation, which combined various deep learning techniques. The COTRNet included U-Net, ResNet, Transformer encoder layer, Pretrained model, and Deep supervision, while still maintaining the original U-shaped architecture. Ultimately, their model achieved the average scores of 0.661 for the dice, and 0.536 for the SD in test set contains 100 CT cases. They did not study the segmentation of cysts and ranked 22nd in KITS21 challenge.

Adam et al. [25] proposed a model with pre-processing, post-processing, and data enhancement functions. They introduced Residual Block in the U-Net structure and achieved 0.951 kidney dice, 0.798 mass dice, 0.781 tumor dice, and an 0.904 kidney SD, 0.648 mass SD, 0.627 tumor SD. The training, validation, and test cases were randomly split into 210, 30, and 60 cases, respectively, out of a total of 300 cases. Their proposed model ResU-Net is similar, and in the end, their results are also similar to 2.5D ResU-Net in Table 2.

Zhao et al. [26] applied the nnU-Net model for coarse-to-fine segmentation of kidney tumors. T They used 20% of the KiTS21 dataset as validation set and their model won first place and outperformed the MFFAU-Net on kidney segmentation with dice and SD. Still, the MFFAU-Net performed better than their model on tumor segmentation. Furthermore, they did not test the cyst segmentation in their study, it is important to note that cysts should not be ignored in kidney tumor segmentation.

Zang et al. [33] used a 3D U-Net with the incorporation of deep supervision, foreground oversampling, and large-scale image context for kidney and tumor segmentation. They also trained their model on majority-predicted segmentation masks. Ultimately, they validated 60 cases and reported a dice of 0.970, 0.851, and 0.819 for kidney, mass, and tumor segmentation. Additionally, they achieved an SD score of 0.937, 0.720, and 0.700 for the same segmentation tasks. 3D U-Net still showed excellent segmentation performance in their study.

Chen et al. [34] employed a cascaded semantic segmentation model in KiTS21. First, they utilized ResSENormUnet for coarse segmentation, which combined SE blocks with normalization to improve the model’s performance. Then, during the fine segmentation, a Transformer was added to the DenseUnet, and DenseTransUnet was constructed to achieve tumor and cyst segmentation. Finally, they validated 60 cases and reported 0.943 kidney dice, 0.778 tumor dice, and 0.710 cyst dice. Their solution needed to construct two different models, and our cascaded segmentation scheme only needed to use the same model.

Li et al. [35] introduced the 3D deep learning for segmentation of kidney. Their method was divided into two stages where the first stage focused on kidney while the second stage focused on tumor and cyst. The architecture of the deep learning network was based on 3D residual U-Net. For validation, they used 30 cases and results were comparable to the ResU-Net and DenseU-Net models presented in Table 2.

George [36] proposed a 3D U-Net network for kidney tumor segmentation. First, the network was trained on down-sampled CT volumes to segment the kidney region. Second, another 3D U-Net was trained on full-resolution images of the kidney region. George reported 0.975 kidney dice, 0.871 tumor dice, and 0.881 cyst dice with 5-fold. Their segmentation results demonstrated the effectiveness of the cascaded 3D U-Net in medical image segmentation. However, 3D models require a lot of computing resources and memory consumption. The cascaded 2.5D model proposed in this paper can achieve similar effects while saving computing resources.

This paper used a cascaded 2.5D MFFAU-Net model to segment kidneys, masses, tumors, and cysts, and good segmentation results were achieved. After analyzing the state-of-the-art methods, it can be concluded that the cascaded segmentation approach is still widely used in medical image segmentation, with the 3D U-Net demonstrating outstanding segmentation performance compared to other models. Despite this, the method presented in this study exhibits superior performance compared to other models when it comes to the segmentation of masses and tumors. Finally, our model attained a remarkable dice score of 0.975 for kidney and 0.875 for tumor in KiTS19, and achieved the average score of 0.875 for dice and 0.794 for SD in KiTS21.

## Conclusion

The 2.5D MFFAU-Net model is the proposed method in this paper for segmenting kidney tumors in 3D images. This model uses 2D convolution to incorporate 3D information in a memory-efficient manner during training. In addition, MFFAU-Net model combined high-level and low-level feature information on the original U-Net structure and introduces the attention gate for improved accuracy in kidney tumor segmentation. Finally, 2.5D MFFAU-Net achieved good segmentation results on both KiTS19 and KiTS21 datasets. In future work, we will also collect and validate more types of images, such as MRI, to develop segmentation approach that can be applied to various image modalities.

## Data Availability

The KiTS19 data can download from https://github.com/neheller/kits19, the KiTS21 data can download from https://github.com/neheller/kits21. All data can be obtained by contacting the first author (Peng Sun, wxsunpeng@163.com).
